# Differences in community awareness regarding the discharge of treated water from the Fukushima Daiichi nuclear power station

**DOI:** 10.1093/jrr/rrae027

**Published:** 2024-05-07

**Authors:** Aizhan Zabirova, Hitomi Matsunaga, Makiko Orita, Yuya Kashiwazaki, Xu Xiao, Noboru Takamura

**Affiliations:** Department of Global Health, Medicine and Welfare, Atomic Bomb Disease Institute, Nagasaki University Graduate School of Biomedical Sciences, Sakamoto 1-12-4, Nagasaki 852-8523, Japan; Department of Global Health, Medicine and Welfare, Atomic Bomb Disease Institute, Nagasaki University Graduate School of Biomedical Sciences, Sakamoto 1-12-4, Nagasaki 852-8523, Japan; Department of Global Health, Medicine and Welfare, Atomic Bomb Disease Institute, Nagasaki University Graduate School of Biomedical Sciences, Sakamoto 1-12-4, Nagasaki 852-8523, Japan; Department of Global Health, Medicine and Welfare, Atomic Bomb Disease Institute, Nagasaki University Graduate School of Biomedical Sciences, Sakamoto 1-12-4, Nagasaki 852-8523, Japan; Department of Global Health, Medicine and Welfare, Atomic Bomb Disease Institute, Nagasaki University Graduate School of Biomedical Sciences, Sakamoto 1-12-4, Nagasaki 852-8523, Japan; Department of Global Health, Medicine and Welfare, Atomic Bomb Disease Institute, Nagasaki University Graduate School of Biomedical Sciences, Sakamoto 1-12-4, Nagasaki 852-8523, Japan

To the Editor,

Thirteen years have passed since the accident at the TEPCO Fukushima Daiichi Nuclear Power Station (FDNPS). During this time, Japanese authorities have implemented and accelerated the decommissioning of FDNPS as reliably and safely as possible [1].

FDNPS consisted of six reactors located on the Pacific coast, and Okuma town contained four of them. Okuma town stores tanks of treated water, totaling >1 million tons, purified through a special system called Advanced Liquid Processing System (ALPS). Tritium remains in the treated water because it cannot be removed by ALPS [2]. The smooth completion of the decommissioning and dismantling process is critical to reconstruction for residents living in Okuma. Meanwhile, the town of Tomioka is located adjacent to Okuma town and is within 20 km from FDNPS (Fig. 1). Tomioka town also faces the Pacific Ocean, and its fishing port was re-opened in 2019, 8 years after the FDNPS accident. Discharge of treated water (DTW) from FDNPS into the Pacific Ocean provoked strong public reaction and concern, including from the international community, and Fukushima’s fishermen [3]. These events interacted with psychological, social and cultural processes that can influence public risk perceptions. Therefore, we investigated whether there were differences in concerns about DTW from FDNPS, risk perceptions regarding genetic effects, and consumption of local food from Okuma or Tomioka approximately half a year before the beginning of DTW into the ocean.

**Fig. 1:**
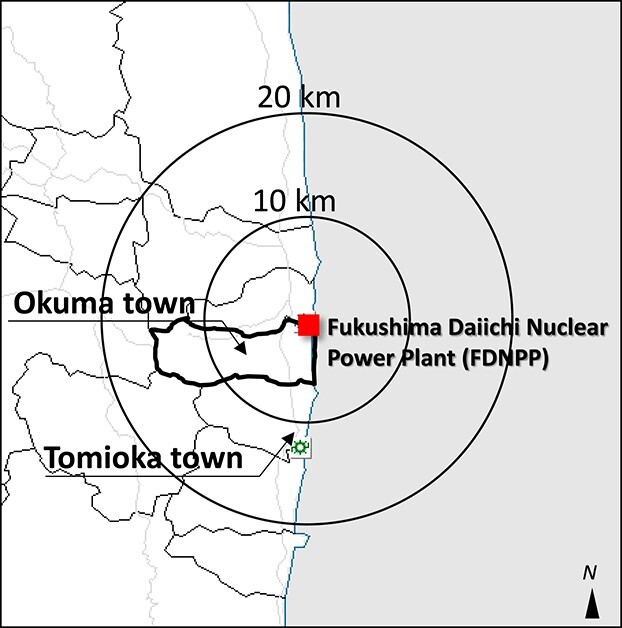
Map of Tomioka and Okuma.

All processes in the study were approved by the ethics committee of the Nagasaki University Graduate School of Biomedical Sciences (No. 21082702). The questionnaire was enclosed in the public relations magazine sent to applicants with a resident card in Tomioka and Okuma in November 2022 using the Basic Resident Ledger in two copies. The period of data collection was from November 2022 to January 2023. The number of responses was 702 and 676 from ~7250 and 4900 households to which each magazine was distributed in Tomioka and Okuma, respectively. Of the total responses, 691 in Tomioka and 669 in Okuma were regarded as valid after excluding incomplete responses. Responses to the questionnaire were requested from those aged >18 years with residential cards in Tomioka or Okuma via paper or a QR code printed in the questionnaire at the time of the survey. We asked about concerns about DTW, ‘Do you have concerns about DTW from FDNPS’, in the questionnaire.

The characteristics of the study participants are shown in Table 1. To compare risk perceptions and anxieties in each town, we performed a binomial logistic regression analysis with ‘yes’ as the reference (Table 2). The proportion of respondents indicating a belief that genetic effects will occur because of the FDNPS accident was lower in Tomioka than in Okuma (odds ratio [OR] = 0.61, 95% confidence interval [95%CI]: 0.49–0.76, *P* < 0.01). Furthermore, reluctance to consume locally grown foods (from Tomioka or Okuma) was lower in Tomioka than in Okuma (OR = 0.77, 95%CI: 0.62–0.95, *P* < 0.05). By contrast, concerns about DTW were not significantly different in Tomioka and Okuma (OR = 1.18, 95%CI: 0.94–1.47, *P* = 0.135). The same as previous studies on radiation exposure [4], females were more concerned about DTW than males (OR = 0.50, 95%CI: 0.40–0.62, *P* < 0.01).

**Table 1:** Characteristics of the study participants

		Overall (*n* = 1360)
		% (n)
Age (years)	< 65	40.2 (547)
	≥ 65	58.6 (797)
	No response	1.2 (16)
Sex	Male	50.0 (681)
	Female	49.3 (670)
	No response	0.7 (9)
Genetic effects will occur because of the FDNPPS accident	Yes	15.1 (206)
	Probably Yes	29.5 (401)
	Probably No	32.3 (439)
	No	21.7 (295)
	No response	1.4 (19)
Reluctant to consume foods from Tomioka/Okuma	Yes	17.2 (235)
	Probably Yes	30.4 (413)
	Probably No	32.9 (447)
	No	18.8 (256)
	No response	0.7 (9)
Concerned about the DTW	Yes	25.4 (346)
	Probably Yes	32.3 (439)
	Probably No	26.3 (357)
	No	15.3 (208)
	No response	0.7 (10)

Note: DTW, discharge of treated water

**Table 2:** Independent differences between Tomioka and Okuma regarding radiation risk perceptions

Variables	Values	Genetic effects will occur because of the FDNPS accident OR (95% CI)	Reluctant to consume foods from Tomioka or Okuma OR (95% CI)	Concerned about the DTW OR (95% CI)
Age	< 65/≥ 65	0.81 (0.64–1.01)	1.04 (0.83–1.30)	0.81 (0.65–1.02)
Sex	Male/Female	0.62 (0.50–0.77)^*^^*^	0.65 (0.53–0.81)^*^^*^	0.50 (0.40–0.62)^*^^*^
Town	Tomioka/Okuma	0.61 (0.49–0.76)^*^^*^	0.77 (0.62–0.95)^*^	1.18 (0.94–1.47)

Note: Binomial logistic regression analysis. Reference for each model is yes. OR, odds ratio; CI, confidence interval; FDNPS, Fukushima Daiichi Nuclear Power Station; DTW, discharge of treated water. *P* < 0.05^*^, *P* < 0.01^*^^*^

There were differences in the towns concerning risk perceptions of radiation and anxiety about foods, although their citizens’ awareness levels of DTW were similar. It is imperative to consider the unique characteristics and sentiments inherent to each society. Factors such as the occupations and cultures specific to each town must be carefully examined and integrated into sustained science-based risk communication.

In August 2023, FDNPS began DTW into the Pacific Ocean. Our study found that ~60% of the residents had concerns about DTW from FDNPS. The International Atomic Energy Agency (IAEA), based on its comprehensive assessment, has concluded that the approach to the discharge of ALPS-treated water into the sea and the associated activities of TEPCO, NRA and the Government of Japan are consistent with relevant international safety standards. Furthermore, the IAEA stated that the radiological impact of DTW on people and the environment will be negligible based on comprehensive scientific evidence [5].

Knowledge of community differences, such as those between Tomioka and Okuma towns, serves as crucial information in understanding the nuanced dynamics surrounding the FDNPS disaster and its aftermath. It is essential in continued efforts to comprehend the social challenges facing the people affected by the nuclear accident.

## CONFLICT OF INTEREST

The authors declare that they have no competing interests.

## Ethical statement

This study was approved by the ethics committee of the Nagasaki University Graduate School of Biomedical Sciences (No. 21082702).

## References

[1] Ministry of the Environment, Japan . Decontamination Projects for Radioactive Contamination Discharged by Tokyo Electric Power Company Fukushima Daiichi Nuclear Power Station Accident. Japan. http://josen.env.go.jp/en/policy_document/pdf/decontamination_projects_1902_01.pdf (23 March 2024, date last accessed).

[2] Ministry of Economy, Trade and Industry (METI) . Basic Policy on Handling of ALPS Treated Water at the Tokyo Electric Power Company Holdings’ Fukushima Daiichi Nuclear Power Station. Japan. https://www.meti.go.jp/english/earthquake/nuclear/decommissioning/pdf/bp_alps.pdf (23 March 2024, date last accessed).

[3] Reuters . Fukushima Water Release Stokes Fresh Fears for Fishermen. The United Kingdom. https://www.reuters.com/investigates/special-report/japan-fukushima-water-fish/ (23 March 2024, date last accessed).

[4] Matsunaga H , Orita M, Taira Y, et al. Intention to return and perception of the health risk due to radiation exposure among residents in Tomioka town, Fukushima prefecture, stratified by gender and generation. Disaster Med Public Health Prep 2020;16:1–8. 33256886 10.1017/dmp.2020.319

[5] International Atomic Energy Agency (IAEA) . IAEA Comprehensive Report on the Safety Review of the ALPS-Treated Water at the Fukushima Daiichi Nuclear Power Station. Republic of Austria. https://www.iaea.org/sites/default/files/iaea_comprehensive_alps_report.pdf (23 March 2024, date last accessed).

